# Respiratory system impedance in different decubitus evaluated by impulse oscillometry in individuals with obesity

**DOI:** 10.1371/journal.pone.0281780

**Published:** 2023-02-14

**Authors:** Mayara Holtz, Larissa Perossi, Jéssica Perossi, Daniele Oliveira dos Santos, Hugo Celso Dutra de Souza, Ada Clarice Gastaldi

**Affiliations:** Health Sciences Department, Physiotherapy Course, Ribeirão Preto Medical School, University of São Paulo, Ribeirão Preto, São Paulo, Brazil; New York University School of Medicine, UNITED STATES

## Abstract

**Background and objective:**

The body posture can influence gas exchange, respiratory mechanics, and mucociliary clearance and different positions can be used as a therapeutic strategy to improve in gas exchange and can also help physiotherapists to assist patients who have difficult or restrictions to stay seated or the ones who stay in the same position for a long period. The objective of this study was to evaluate the effect of different positions on respiratory system impedance in obese and eutrophic subjects, using Impulse Oscillometry System (IOS).

**Methods:**

The IOS parameters were evaluated in seated (Se), right lateral decubitus (RL), left lateral decubitus (LL), and supine (Su).

**Results:**

Sixty two volunteers were allocated in obese group (OG) or eutrophic group (EG) according to BMI. In seated position, OG showed higher impedance than EG for R5: 0.55 (0.31; 0.93) and 0.33 (0.24; 0.52); R20: 0.39 (0.23; 0.54) and 0.32 (0.03; 0.41); R5-R20: 0.13 (0.02; 0.47) and 0.01 (-0.08; 0.27); X5: -0.20 (-0.51; 0.16) and -0,10 (-0.016; -0.04); Fres: 20.59 (11.54; 36.45 and 10.69 (7.56; 24.7) (p<0.05) and the impedance were higher in the Su for both groups. Compared to Se, there were differences with Su (R5, R5-20, X5), with RL (R20), and with LL (R5, R20) for OG; and with Su (R5, R5-20, X5, Fres), with RL and LL (X5) for EG. Compared to Su, there were differences with RL and LL (R5-20, X5) for OG; and with RL (R5, R5-20, X5, Fres), and LL (R5-20, X5, Fres) for EG. There were no differences between RL and LL for OG and EG.

**Conclusion:**

The respiratory system impedance is increased in OG, with greater contribution of peripheral resistance. The higher values of resistance and reactance were obtained in the supine position, in both groups, with lower differences obtained in the right and left lateral decubitus.

## Introduction

For a good functioning of the respiratory system, a synchronism between rib cage and pulmonary structures is required. These structures work closely with each other and can be affected and influenced by the posture, through the changes of gravity force action [1]. The influence of body posture on distribution of ventilation and perfusion is already well established, with an increase in the gravity dependent areas. The posture can also influence pulmonary function, but some questions are controversial and need to be answered [2–5, 6].

Different positions can be used as a therapeutic strategy, such as the prone position in acute respiratory distress syndrome and coronavirus (COVID-19) patients, aiming an improvement in gas exchange [4, 5]. Postural changes can also help physiotherapists to assist patients who have difficulty or restrictions to stay seated or the ones who stay in the same position for long periods, such as wheelchair users, bedridden patients, pregnant, elderly and people with obesity [7].

Eutrophic subjects may have a decrease in the pulmonary volumes in the lying position, and subjects with obesity, due to the thoracoabdominal deposition of adipose tissue, have an additional decrease in diaphragmatic mobility, leading to hypoventilation and an increase in the work of breathing, even in the seated position. It is still unclear whether changes in posture can induce changes in the elastic properties of the lungs in obese and if they are similar or not to eutrophic individuals [8–10].

Considering that the impulse oscillometry system (IOS) has potential utility to identify pathological changes in obesity, even with normal spirometry, and that it has been well studied in seated or supine [11] obese volunteers, the objective of this study was to evaluate the effect of different positions on respiratory system impedance of obese volunteers.

## Methods

This is a cross-sectional study developed between August 2015 and October 2016 at Ribeirão Preto Medical School (FMRP), University of São Paulo (USP), in the Laboratory for Assessment of Respiratory System (LAR). It was approved by the local Human Research Ethics Committee (CAAE n° 34717314.5.0000.5440), and all patients signed the Informed Consent Form.

The sample size was calculated using the GPower software (version 3.1.9.2), based on the study of Behrakis et al. (1983), based on the airways resistance variable, with a difference of 0.7 cmH_2_O, standard deviation of 0.13 cmH_2_O, α of 5% and power of 90. The calculation resulted in a sample of 36 patients [3].

Volunteers were enrolled from the community and from Bariatric Surgery Ambulatory of FMRP-USP tertiary Hospital, aged between 18 and 50 years old and without associated uncontrolled comorbidities (hypertension, diabetes or dyslipidemia). They were allocated in 2 groups according to body mass index (BMI), the obese group (OG) with BMI ≥ 40kg/m^2^ and the eutrophic group (EG) with BMI between 18.5 and 24.9 kg/m^2^. Subjects with obstructive and restrictive pulmonary diseases, active smokers, and cardiovascular conditions, neuromuscular or musculoskeletal diseases which compromised the execution of the protocol were excluded.

After recruitment, the participant was invited to participate in the evaluation protocol. Anthropometric data including age, sex, weight and height was registered, the respiratory impedance evaluation was performed through IOS in different decubitus and the pulmonary function evaluation was performed by spirometric test in seated position. Spirometry and IOS evaluations were conducted by trained professionals in controlled environments during the day, with daily equipment verification to ensure the accuracy of the tests.

### Impulse oscillometry system

We used the Jaeger IOS equipment (Jaeger, Wurzburg, Germany) to evaluate the respiratory system impedance which includes respiratory resistance and reactance, parameters that provide information about the mechanical properties of the airways and lung parenchyma during spontaneous breathing [11–13]. The tests were performed in a temperature-controlled room (73.4°F).

IOS evaluation was conducted before any other tests, according to Oostveen et al. (2003) and King et al. (2020) recommendations, with the subjects wearing a nose clip, hands supporting the cheeks to decrease their oscillation during the procedure, and lips tightly sealed around the mouthpiece to avoid air leaks [12, 14]. A free-flow mouthpiece was used in order to stabilize the position of their tongue [15]. The predicted values were calculated from Vogel & Smith (1994) [16].

The volunteer was instructed to perform the IOS test at four positions: seated (Se); supine (Su); right lateral decubitus (RL); and left lateral decubitus (LL). The first position was determined by chance and followed by the others: Se-Su-RL-LL, or Su-RL-LL-Se, or RL-LL-Se-Su, or LL-Se-Su-RL. The volunteer adopted each position after finishing the previous one, stayed for five minutes of rest, and performed the measurements. For each lateral decubitus a pillow was used to support the head in a neutral position.

We analyzed the impedance parameters of whole-breath at 5 and 20 Hz (R5, R20 and X5), the frequency dependence of the respiratory system resistance (Rrs) (R5-R20) and the resonant frequency (Fres).

### Spirometry

We used the Koko PFT System (version 4.11, 2007 nSpire Health Inc.; Pulmonary Data Services, United States) to evaluate the pulmonary function of the participants. The test was performed in seated position, after the IOS exams once spirometry requires effort-dependent maneuvers. The Brazilian Guidelines for Pulmonary Function Tests was followed during the test performance and the equation proposed by Crapo et al. (1981) was used to calculate the predicted values of the variables (Forced vital capacity–FVC; forced expiratory volume in one second—FEV_1;_ FEV_1_/FVC and mean forced expiratory flow—FEF_25–75%_) [17, 18].

### Statistical analysis

The software SPSS (version 22.0, IBM Corporation, 2013) was used. Data distribution was tested using the Shapiro-Wilk test. The Mann-Whitney Test was used to compare the characterization of the participants and the demographic data of OG and EG and to compare the IOS values in each different decubitus of OG and EG. The Friedman Test was used to compare the IOS values in different decubitus for each group. The results were considered significant with *p* < 0.05.

## Results

Sixty-two participants were selected for the present study. For the spirometry test, we compared the percentage of predicted values and there was no difference between the EG and OG groups. Participant’s characterization data is demonstrated in **Table 1**.

**Table 1:** Characterization data of the participants.

	EG (n = 28)	OG (n = 34)	*P*
**Sex (W/M)**	27/1	31/3	-
**Age (years)**	26 (21, 39)	34.5 (22, 53)	<0.0001
**Weight (kg)**	61.4 (49, 77.1)	130.7 (98, 182)	<0.0001
**Height (m)**	1.67 (1.53, 1.84)	1.63 (1.49, 1.84)	0.193
**BMI (kg/m** ^**2**^ **)**	21.79 (17.16, 25.00)	49.01 (40.35, 61.69)	<0.0001
**%FVC**	96 (81, 113)	97.5 (57, 115)	0.599
**%FEV**_**1**_	95.5 (83, 115)	91 (54, 115)	0.106
**%FEV**_**1**_ **/FVC**	100 (87, 110)	98 (83, 108)	0.181
**%FEF**_**25-75**_	90.5 (63, 127)	85.5 (24, 130)	0.221

EG: eutrophic group; OG: obesity group; W: women; M: men; H/W R: hip waist ratio; FVC: forced vital capacity; FEV_1_: forced expiratory volume in the first second; FEF_25-75%_: mean forced expiratory flow

The comparison of IOS parameters between OG and EG in the same position, showed that OG values were significantly higher than those for EG for all positions and all IOS parameters (**Table 2**).

**Table 2:** Comparison of IOS parameters between EG and OG groups.

	OG	EG	*p*
**Predicted**			
R5	0.35 (0.26; 0.38)	0.34 (0.26; 0.36)	0,005
R20	0.29 (0.22; 0.33)	0.28 (0.22; 0.30)	0,003
R5-R20	0.06 (0.01; 0.06)	0.06 (0.04; 0.06)	0,236
X5	-0.02 (-0.07; 0.2)	0.00 (-0.03; 0.02)	0,003
**Se**			
R5	0.55 (0.31; 0.93)	0.33 (0.24; 0.52)	<0,0001
R20	0.39 (0.23; 0.54)	0.32 (0.03; 0.41)	<0,0001
R5-R20	0.13 (0.02;0.47)	0.01 (-0.08; 0.27)	<0,0001
X5	-0.20 (-0.51; 0.16)	-0.10 (-0.16; -0.04)	<0,0001
Fres	20.59 (11.54; 36.45)	10.69 (7.56; 24.70)	<0,0001
**RL**			
R5	0.55 (0.36; 0.97)	0.35 (0.23; 0.53)	<0,0001
R20	0.42 (0.29; 0.64)	0.35 (0.27; 0.48)	<0,0001
R5-R20	0.13 (0.03; 0.40)	0.00 (-0.04; 0.14)	<0,0001
X5	-0.22 (-0.54; -0.12)	-0.12 (-0.19; -0.08)	<0,0001
Fres	18.65 (12.46; 34.99)	10.73 (8.35; 21.65)	<0,0001
**LL**			
R5	0.55 (0.33; 0.96)	0.35 (0.25; 0.52)	<0,0001
R20	0.44 (0.27; 0.57)	0.33 (0.24; 0.43)	<0,0001
R5-R20	0.12 (0.06; 0.39)	0.01 (-0.04; 0.16)	<0,0001
X5	-0.23 (-0.50; -0.13)	-0.12 (-0.20; -0.07)	<0,0001
Fres	19.68 (13.33; 37.10)	10.85 (8.61; 19.59)	<0,0001
**Su**			
R5	0.60 (0.36; 0.95)	0.37 (0.26; 0.51)	<0,0001
R20	0.42 (0.30; 0.55)	0.32 (0.26; 0.42)	<0,0001
R5-R20	0.15 (0.04; 0.40)	0.04 (-0.01; 0.17)	<0,0001
X5	-0.28 (-0.66; -0.14)	-0.15 (-0.20; -0.08)	<0,0001
Fres	20.43 (11.07; 36.11)	14.44 (9.85; 23.73)	<0,0001

Data expressed in Median (Minimum; Maximum)

R5: total respiratory system resistance; R20: central airways resistance; R5-R20: peripheral airways resistance; X5: respiratory system reactance; Fres: resonant frequency; Se: seated; RL: right lateral decubitus; LL: left lateral decubitus; Su: supine position.

**Fig 1:**
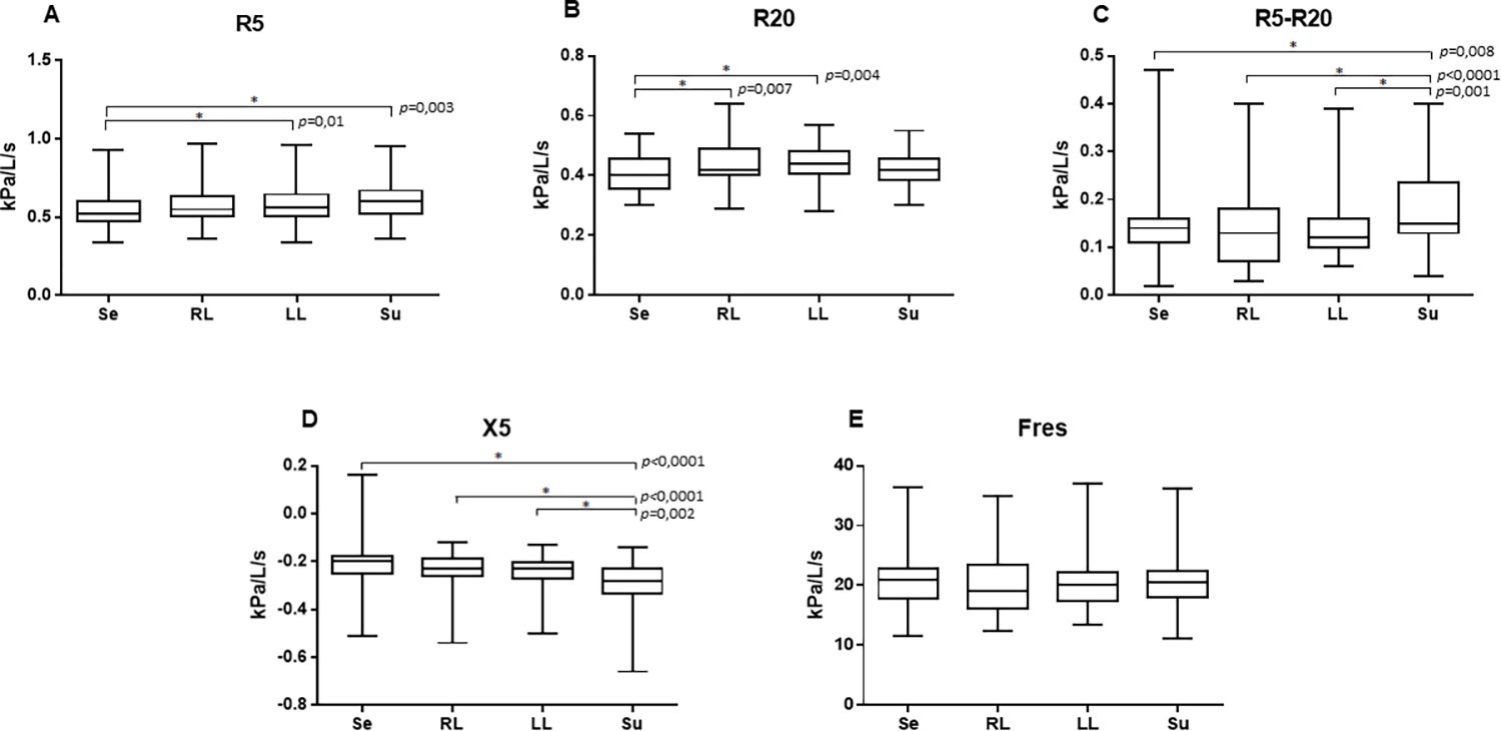
Graphs with comparisons between postures for obese group. (A) R5, (B) R20, (C) R5-R20, (D) X5, (E) Fres. *p<0.05. Se: seated; RL: right lateral decubitus; LL: left lateral decubitus, Su: supine. R5: total respiratory system resistance; R20: central airways resistance; R5-R20: frequency dependence of the respiratory system resistance; X5: respiratory system reactance; Fres: resonant frequency.

In OG, for R5, Se (0.55 (0.31; 0.93) Hz) was different from LL (0.55 (0.33; 0.96) Hz;) and Su (0.60 (0.36; 0.95) Hz). For R20, Se (0.39 (0.23; 0.54) Hz) was different from RL (0.42 (0.29; 0.64) Hz) and LL (0.44 (0.27; 0.57) Hz). For R5-R20, Su (0.15 (0.04; 0.40) Hz) was higher than RL (0.13 (0.03; 0.40) Hz) and LL (0.12 (0.06; 0.39) Hz) and; for X5, Su was more negative (-0.28 (-0.66; -0.14) Hz) than RL (-0.22 (-0.54; -0.12) Hz) and Se (-0.20 (-0.51; 0.16) Hz) (**Fig 1**).

**Fig 2:**
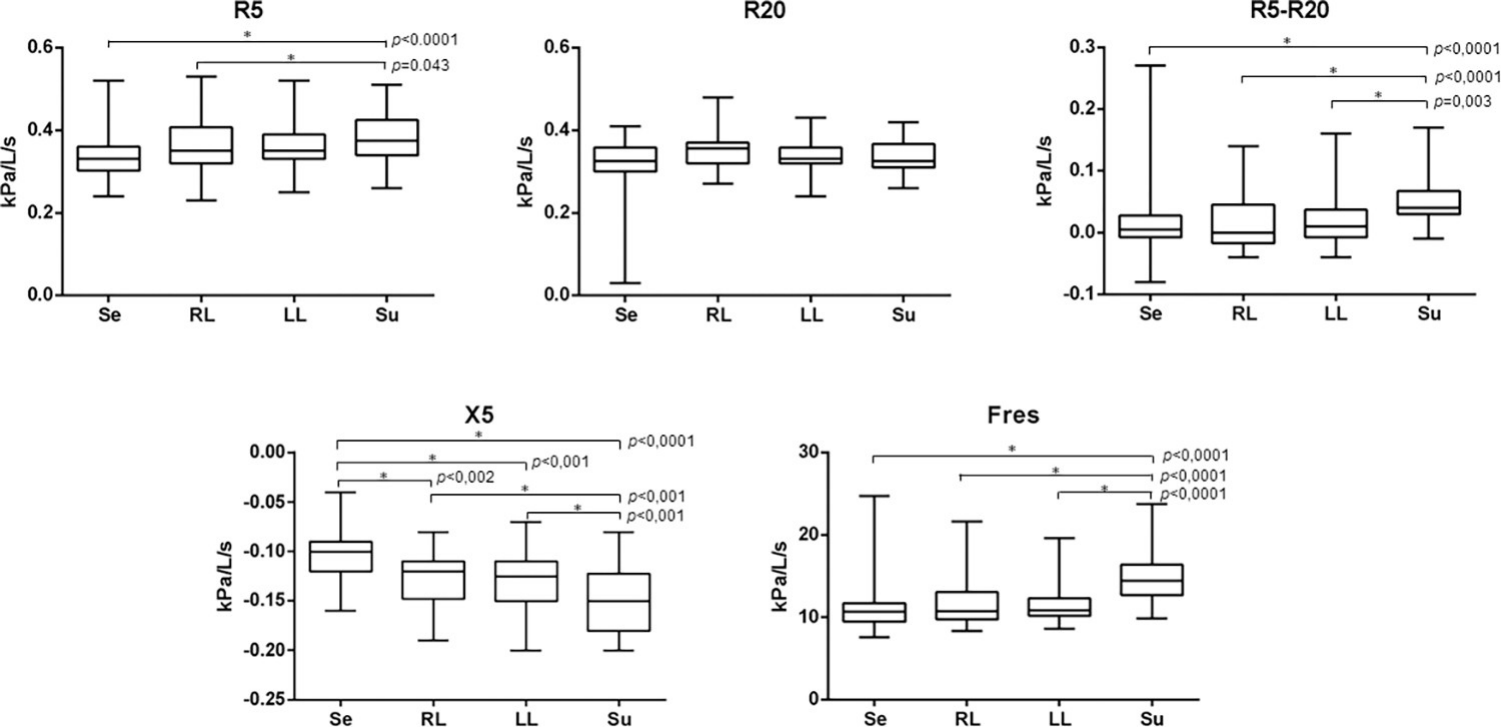
Graphs with comparisons between postures for eutrophic group. (A) R5 (B) R20, (C) R5-R20, (D) X5 and (E) Fres. *p<0.05. Se: seated; RL: right lateral decubitus; LL: left lateral decubitus; Su: supine. R5: total respiratory system resistance; R20: central airways resistance; R5-R20: frequency dependence of the respiratory system resistance; X5: respiratory system reactance; Fres: resonant frequency.

In EG there were differences between decubitus for all parameters and, Su presented statistically higher values of resistance (R5: 0.37 (0.26; 0.51) Hz; R5-20: 0.04 (-0.01; 0.17) Hz) and more negative of reactance (X5: -0.15 (-0.20; -0.08) Hz) for all parameters except in R20, in which RL was greater than Se and Su, without difference with LL (**Fig 2**).

## Discussion

This study evaluated the total impedance of respiratory system in obese and eutrophic subjects at different positions and the results showed higher impedance in OG compared to EG for all positions. The higher values of resistance and reactance were obtained in the supine position, in both groups, with lower differences obtained in the right and left lateral decubitus compared to seated. The OG had changes in the R20 in right and left lateral decubitus, that was not observed in the EG.

It was demonstrated that in grade III obesity subjects, airway resistance was 56% higher than those with grade I, with correlation between the flow-volume curve and the resistance, indicating an obstruction and/or air trapping in peripheral airways. However, conductance of the airways did not change as the BMI increases, suggesting that this increase in resistance may be related to lung volume [19]. Confirming these results, in the present study, the OG, within normal spirometry limits, had higher airways resistance values than EG, being the R5 61% higher, provided by a compartmentalized IOS analysis.

In non-obese subjects, there is a reduction in FRC and an increase in resistance when adopting supine posture, attributed to gravitational effects on abdominal contents, resulting in a relaxed and expiratory position of the diaphragm [20–24]. Similar alterations were described for non-obese subjects analyzing FVC, FEV1, and peak expiratory flow (PEF) [25]. Other study comparing healthy and non-obese individuals, found higher values of resistance in the supine position compared to sitting [26].

Spirometry is the most commonly used method for pulmonary function evaluation, recommended to be performed in the seated position, while the PEF is recommended in the seated or stand position [27], but there are controversial results comparing seated and standing positions in subjects with obesity. Domingos-Benício et al. (2004), evaluating groups with different BMI ranges, obtained values within normal range; Berntsen et al. (2011) found higher values in the sitting position in children with obesity; while Gudmundsson et al. (1997) demonstrated larger values in the standing position [8, 28, 29]. However, Miller et al. (2005) found that individuals with obesity are able to inhale more deeply into the standing position, and, consequently, forced expiratory volumes and flows tend to be larger at this position, while eutrophic individuals generally have similar results at both positions [30].

Some studies have demonstrated that the variation in TLC from seated to supine was lower in obese when compared to eutrophic subjects, that can be explained by respiratory system restriction, causing lower decrease in the supine FRC, which limits greater volume variations [19, 20, 31, 32]. In this study, subjects with obesity had higher gravitational effects with posture changes compared to the eutrophic ones, presenting more negative values of total reactance (X5) and higher values of resistance (R5, R20 and R5-R20) in the supine position compared to the sitting, while in the EG these changes were smaller, but Fres were also affected.

Other studies from our group showed a decrease in PEF values in supine and right lateral decubitus, with no difference in relation to left lateral decubitus for eutrophic and obese subjects [33, 34], that is similar to the other authors, that also observed a decrease in right lateral values [32, 35, 36]. In this study, using IOS, no difference was found between RL and LL for any studied parameters, both for eutrophic and subjects with obesity. However, in the comparison between supine and lateral decubitus of OG, it was observed that the supine position showed increased R5-R20 and more negative X5 than RL and LL. Furthermore, it could be observed a higher R20 in LL. It suggests that, in the clinical setting, when vertical or raised position is not possible, lateral decubitus can be an option to reduce airways resistance caused by supine position, with a smaller advantage to RL than LL decubitus.

Because posture changes are commonly used in clinical practice as a strategy for lung expansion, the postures that result in lower respiratory system resistance can produce best flows with less respiratory work [5, 37], which is also important to an effective cough flow. In addition, postural changes can improve gas exchange, and the lateral decubitus can contribute to decreased respiratory loads. The higher baseline impedance and minor variations related to the posture presented by the obese group, may induce respiratory complications when the ventilatory demand increases, as the development of severe acute respiratory syndrome in obese patients with COVID-19 [38].

It is important to note that, because we do not have reference values for Brazilian adults, this study have the eutrophic group measurements as a reference. The strength of these results is the negative effect of supine position in the respiratory mechanics, especially if it takes long periods. Our main limitation was the absence of Z (impedance) and AX (reactance area) values, that is potentially more sensitive to changes in the elastic properties of the respiratory system than reactance at a single frequency. Also, we have had a greater number of women volunteers in this study, which may limit the generalizability of our results.

So, we concluded that there is a higher total impedance of the respiratory system in subjects with obesity, with greater contribution of frequency dependent resistance. The higher values of resistance and reactance were obtained in the supine position, in both groups, with lower differences obtained in the right and left lateral decubitus. In clinical practice, when it is not possible the thorax vertical position (seated or stand), lateral decubitus can be an alternative to reduce the airways resistance promoted by supine position, in eutrophic and subjects with obesity.

## Supporting information

S1 Table Anthropometric, demographic and pumonary function data of 34 obese participants. (PDF)

S2 Table Anthropometric, demographic and pumonary function data of 28 eutrophic participants. (PDF)

S3 Table IOS values of 34 obese participants in different decubitus. (PDF)

S4 Table IOS values of 28 eutrophic participants in different decubitus. (PDF)
