# Supplementary material for: Respiratory system impedance in different decubitus evaluated by impulse oscillometry in individuals with obesity
Source: PLoS One. 2023 Feb 14;18(2):e0281780. doi: 10.1371/journal.pone.0281780 (PMC9928067; doi:10.1371/journal.pone.0281780)
Supplement: S1 Table — (PDF) [file pone.0281780.s001.pdf]

Table S1. Anthropometric, demographic and pumonary function data of 34 obese participants.

Obese Group								
**Age**	**Gender**	**Weight (kg)**	**Height (cm)**	**BMI (kg/m2)**	**%VFC**	**%FEV1**	**%FEV1/CVF**	**%FEF25-75**
37	woman	113.6	159	44.93	90	89	103	92
33	man	151.2	182	45.65	NA	NA	NA	NA
39	woman	148.0	179	46.19	102	92	91	67
42	woman	113.9	162	43.4	103	106	104	120
53	woman	117.3	149	52.84	66	54	83	24
28	man	122.6	172	41.44	93,4	89,0	95,0	79
22	woman	131.8	176	42.55	113	105	92	86
41	woman	144.4	153	61.69	90	83	93	64
44	woman	132.6	165	48.71	86	87	102	109
35	woman	107.0	160	41.8	97	86	88	57
29	woman	135.8	172	45.9	111	104	93	87
42	woman	122.0	156	50.13	102	110	108	105
24	woman	138.8	162	52.89	115,0	115,0	100,0	127
44	woman	120.0	156	49.31	61	58	96	49
28	woman	156.6	167	56.12	93	83	89	57
32	woman	145.0	157	58.83	72	77	107	80
42	woman	120.0	153	51.39	98	89	91	62
43	woman	136.0	162	51.82	98	98	99	89
50	woman	115.4	165	42.39	82	86	106	110
27	woman	129.6	171	44.32	101	103	101	117
34	woman	133.0	157	53.96	109	113	104	130
44	woman	141.7	164	52.68	90	87	97	77
29	woman	142.1	169	49.75	104	89	85	57
33	woman	105.9	158	42.42	91	94	103	115
28	man	169.7	184	50.12	NA	NA	NA	NA
31	woman	123.3	158	49.39	99	93	93	76
48	woman	126.0	167	45.18	108	107	100	100
38	woman	98.0	153	41.86	87	81	93	63
23	woman	148.0	166	53.71	98	92	93	84
42	woman	134.0	167	48.08	94	98	105	115
24	woman	124.2	165	45.62	103	99	95	85
44	woman	102.0	159	40.35	90	90	101	93
25	woman	125.0	159	49.44	57	58	103	70
28	woman	182.0	175	59.43	101	101	99	95
