# Supplementary material for: Respiratory system impedance in different decubitus evaluated by impulse oscillometry in individuals with obesity
Source: PLoS One. 2023 Feb 14;18(2):e0281780. doi: 10.1371/journal.pone.0281780 (PMC9928067; doi:10.1371/journal.pone.0281780)
Supplement: S2 Table — (PDF) [file pone.0281780.s002.pdf]

Table S2. Anthropometric, demographic and pumonary function data of 28 eutrophic participants.

Eutrophic Group								
**Age**	**Gender**	**Weight (kg)**	**Height (cm)**	**BMI (kg/m2)**	**%VFC**	**%FEV1**	**%FEV1/CVF**	**%FEF25-75**
22	woman	71.0	172	24.0	97	95	97	79
26	woman	50.3	167	18.0	81	88	107	91
23	woman	60.0	161	23.2	105	107	101	97
24	woman	72.0	170	24.9	92	94	101	99
24	woman	54.4	155	22.6	91	90	98	98
27	woman	71.7	174	23.7	110	102	93	87
29	woman	66.7	166	24.2	104	96	92	77
28	woman	50.0	160	19.5	84	83	99	71
39	woman	58.5	169	20.5	113	115	102	119
24	woman	50.1	158	20.1	103	91	87	63
26	woman	49.6	170	17.2	83	89	107	93
25	woman	71.8	184	21.2	91	91	100	99
26	woman	52.0	156	21.4	99	104	105	113
25	woman	49.0	153	21.0	109	99	91	72
38	woman	62.7	169	22.0	112	105	94	85
27	man	72.6	183	21.7	92	94	100	85
21	woman	62.0	172	21.0	94	94	99	90
24	woman	64.0	160	24.9	98	107	108	125
29	woman	70.0	178	22.1	100	100	100	90
34	woman	51.0	156	21.0	91	86	94	73
25	woman	56.0	159	22.2	85	86	102	89
25	woman	53.0	160	20.7	88	97	110	113
29	woman	61.0	167	21.9	91	93	101	111
32	woman	59.1	165	21.7	102	96	94	74
39	woman	66.0	173	22.0	112	112	101	100
28	woman	61.8	171	21.1	90	89	99	84
33	woman	64.8	162	24.7	107	112	104	127
23	woman	77.1	181	23.5	95	101	107	102
