# Supplementary material for: Respiratory system impedance in different decubitus evaluated by impulse oscillometry in individuals with obesity
Source: PLoS One. 2023 Feb 14;18(2):e0281780. doi: 10.1371/journal.pone.0281780 (PMC9928067; doi:10.1371/journal.pone.0281780)
Supplement: S3 Table — (PDF) [file pone.0281780.s003.pdf]

Table S3. IOS values of 34 obese participants in different decubitus.

Predicted values				Seated position					Right lateral decubitus					Left lateral decubitus					Supine position				
R5	X5	R20	R5-R20	R5	X5	R20	R5-R20	Fres	R5	X5	R20	R5-R20	Fres	R5	X5	R20	R5-R20	Fres	R5	X5	R20	R5-R20	Fres
0.36	-0.03	0.3	0.06	0.43	-0.22	0.36	0.07	16.45	0.53	-0.21	0.46	0.07	15.04	0.51	-0.22	0.44	0.07	17.15	0.6	-0.28	0.45	0.15	19.15
0.27	0.02	0.23	0.04	0.51	-0.14	0.4	0.11	17.59	0.46	-0.12	0.41	0.05	12.92	0.55	-0.13	0.43	0.12	16.07	0.38	-0.14	0.3	0.08	12.44
0.36	-0.03	0.3	0.06	0.6	-0.26	0.41	0.19	22.31	0.65	-0.24	0.47	0.18	20.4	0.53	-0.23	0.41	0.12	21.31	0.69	-0.4	0.41	0.28	22.08
0.36	-0.04	0.3	0.06	0.47	-0.18	0.42	0.05	16.44	0.48	-0.19	0.41	0.07	18.09	0.52	-0.21	0.42	0.1	17.74	0.5	-0.23	0.41	0.09	20.15
0.38	-0.07	0.32	0.06	0.93	-0.36	0.46	0.47	36.45	0.69	-0.28	0.46	0.23	32.43	0.67	-0.37	0.43	0.24	33.49	0.76	-0.4	0.47	0.29	27.93
0.26	0.02	0.22	0.04	0.52	-0.22	0.39	0.13	24.05	0.58	-0.23	0.48	0.1	22.94	0.56	-0.25	0.41	0.15	23.78	0.58	-0.32	0.44	0.14	22.83
0.33	0.01	0.27	0.06	0.53	-0.13	0.45	0.08	17.9	0.51	-0.16	0.38	0.13	19.02	0.55	-0.18	0.38	0.17	18.03	0.59	-0.19	0.46	0.13	17.23
0.36	-0.04	0.3	0.06	0.64	-0.3	0.46	0.18	22.68	0.55	-0.27	0.39	0.16	19.63	0.69	-0.32	0.53	0.16	26.29	0.6	-0.35	0.42	0.18	20.87
0.37	-0.04	0.31	0.06	0.47	-0.09	0.31	0.16	16.7	0.52	-0.2	0.4	0.12	16.67	0.49	-0.2	0.39	0.1	16.57	0.6	-0.28	0.35	0.25	20.64
0.35	-0.02	0.29	0.06	0.61	-0.19	0.45	0.16	24.73	0.59	-0.21	0.46	0.13	24.01	0.61	-0.25	0.46	0.15	25.8	0.59	-0.28	0.4	0.19	23.28
0.34	-0.01	0.28	0.06	0.66	-0.19	0.54	0.12	21.81	0.7	-0.24	0.64	0.06	24.93	0.52	-0.18	0.45	0.07	19.37	0.62	-0.21	0.52	0.1	15.99
0.36	-0.04	0.3	0.06	0.48	-0.04	0.37	0.11	20.97	0.55	-0.26	0.49	0.06	18.28	0.59	-0.27	0.47	0.12	21.18	0.51	-0.24	0.39	0.12	19.35
0.33	0.001	0.27	0.06	0.39	-0.17	0.3	0.09	15.24	0.51	-0.16	0.42	0.09	14.61	0.53	-0.16	0.45	0.08	14.34	0.5	-0.21	0.37	0.13	16.27
0.37	-0.04	0.31	0.06	0.84	-0.51	0.5	0.34	34.07	0.9	-0.54	0.5	0.4	34.99	0.96	-0.48	0.57	0.39	37.1	0.95	-0.56	0.55	0.4	36.11
0.34	-0.01	0.28	0.06	0.5	-0.24	0.35	0.15	21.85	0.62	-0.27	0.42	0.2	25.43	0.54	-0.24	0.44	0.1	21.36	0.53	-0.24	0.38	0.15	20.47
0.35	-0.02	0.29	0.06	0.58	-0.24	0.38	0.2	21.18	0.97	-0.49	0.62	0.35	22.81	0.82	-0.5	0.49	0.33	22.39	0.81	-0.66	0.45	0.36	27.27
0.36	-0.04	0.3	0.06	0.67	-0.31	0.46	0.21	26.96	0.79	-0.3	0.57	0.22	23.31	0.72	-0.28	0.57	0.15	20,00	0.72	-0.36	0.43	0.29	23.41
0.37	-0.04	0.31	0.06	0.78	-0.35	0.5	0.28	29.76	0.76	-0.35	0.5	0.26	24.7	0.8	-0.3	0.52	0.28	26.9	0.84	-0.5	0.52	0.32	30.21
0.38	-0.06	0.32	0.06	0.54	-0.34	0.39	0.15	20.84	0.55	-0.24	0.43	0.12	20.4	0.58	-0.27	0.47	0.11	21.89	0.52	-0.24	0.36	0.16	21.6
0.34	-0.001	0.28	0.06	0.34	-0.18	0.32	0.02	11.54	0.36	-0.16	0.29	0.07	13.74	0.34	-0.18	0.28	0.06	13.33	0.36	-0.15	0.32	0.04	11.07
0.35	-0.02	0.29	0.06	0.54	-0.2	0.41	0.13	18.94	0.47	-0.19	0.4	0.07	14.61	0.51	-0.21	0.4	0.11	17.91	0.52	-0.23	0.41	0.11	16.07
0.37	-0.04	0.31	0.06	0.6	-0.23	0.48	0.12	22.15	0.55	-0.13	0.52	0.03	12.46	0.62	-0.22	0.53	0.09	18.84	0.65	-0.26	0.51	0.14	19.28
0.34	-0.01	0.28	0.06	0.5	-0.2	0.37	0.13	18.38	0.57	-0.25	0.42	0.15	16.37	0.68	-0.25	0.56	0.12	21.23	0.67	-0.31	0.52	0.15	18.82
0.35	-0.02	0.29	0.06	0.54	-0.19	0.45	0.09	16.86	0.54	-0.22	0.4	0.14	17.3	0.6	-0.25	0.45	0.15	18.99	0.6	-0.26	0.43	0.17	20.43
0.26	0.02	0.22	0.04	0.49	-0.2	0.35	0.14	20.2	0.5	-0.24	0.37	0.13	16.93	0.64	-0.26	0.46	0.18	20.14	0.49	-0.21	0.38	0.11	16.92
0.35	-0.01	0.29	0.06	0.51	-0.16	0.4	0.11	18.17	0.48	-0.17	0.39	0.09	14.81	0.5	-0.22	0.4	0.1	16.1	0.53	-0.22	0.39	0.14	18.17
0.37	-0.05	0.31	0.06	0.47	-0.19	0.35	0.12	18.69	0.55	-0.22	0.41	0.14	18.16	0.49	-0.23	0.39	0.1	17.98	0.53	-0.27	0.39	0.14	17.89
0.36	-0.03	0.3	0.06	0.51	-0.28	0.37	0.14	22.85	0.58	-0.26	0.45	0.13	19.57	0.51	-0.22	0.42	0.09	17.16	0.59	-0.34	0.4	0.19	21.11
0.33	0.01	0.27	0.06	0.6	-0.18	0.44	0.16	22.89	0.68	-0.23	0.49	0.19	23.57	0.6	-0.23	0.44	0.16	21.14	0.67	-0.26	0.45	0.22	21.16
0.36	-0.04	0.3	0.06	0.64	-0.23	0.5	0.14	20.96	0.62	-0.21	0.57	0.05	15.72	0.65	-0.28	0.51	0.14	21.3	0.62	-0.3	0.49	0.13	18.22
0.33	0,00	0.27	0.06	0.46	0.16	0.36	0.1	15.79	0.47	-0.17	0.4	0.07	18.06	0.48	-0.16	0.39	0.09	16.54	0.63	-0.33	0.44	0.19	21.98
0.37	-0.04	0.31	0.06	0.31	-0.17	0.23	0.08	16.48	0.42	-0.22	0.32	0.1	15.99	0.33	-0.2	0.27	0.06	14.16	NA	NA	NA	NA	NA
0.34	0,00	0.28	0.06	0.44	-0.2	0.3	0.14	19.03	0.57	-0.25	0.39	0.18	19.49	0.48	-0.26	0.32	0.16	17.77	0.56	-0.28	0.36	0.2	19.37
0.34	-0.01	0.33	0.01	0.48	-0.15	0.33	0.15	20.35	0.58	-0.2	0.41	0.17	23.88	0.61	-0.14	0.46	0.15	23.62	0.7	-0.31	0.42	0.28	23.6

R5: total respiratory system resistance; **R20:** central airways resistance; **R5-R20**: peripheral airways resistance; **X5**: respiratory system reactance; **Fres:** resonant frequency.
