# Supplementary material for: Respiratory system impedance in different decubitus evaluated by impulse oscillometry in individuals with obesity
Source: PLoS One. 2023 Feb 14;18(2):e0281780. doi: 10.1371/journal.pone.0281780 (PMC9928067; doi:10.1371/journal.pone.0281780)
Supplement: S4 Table — (PDF) [file pone.0281780.s004.pdf]

Table S4. IOS values of 28 eutrophic participants in different decubitus.

Predicted values				Seated position					Right lateral decubitus					Left lateral decubitus					Supine position				
R5	X5	R20	R5-R20	R5	X5	R20	R5-R20	Fres	R5	X5	R20	R5-R20	Fres	R5	X5	R20	R5-R20	Fres	R5	X5	R20	R5-R20	Fres
0.33	0.01	0.27	0.06	0.33	-0.09	0.3	0.03	11.39	0.33	-0.11	0.28	0.05	13.43	0.35	-0.11	0.27	0.08	13.51	0.37	-0.13	0.3	0.07	14.65
0.34	-0.001	0.28	0.06	0.35	-0.13	0.33	0.02	12.38	0.41	-0.16	0.42	-0.01	11.4	0.41	-0.15	0.42	-0.01	11.39	0.38	-0.18	0.34	0.04	16.5
0.33	0.01	0.27	0.06	0.32	-0.09	0.33	-0.01	9.2	0.35	-0.12	0.38	-0.03	9.45	0.36	-0.12	0.33	0.03	10.98	0.36	-0.14	0.32	0.04	13.46
0.33	0.001	0.27	0.06	0.33	-0.12	0.3	0.03	11.31	0.32	-0.14	0.32	0.00	10.91	0.33	-0.13	0.33	0.00	10.87	0.34	-0.15	0.29	0.05	14.66
0.33	0.001	0.27	0.06	0.3	-0.15	0.28	0.02	13.29	0.32	-0.18	0.27	0.05	13.14	0.27	-0.17	0.24	0.03	13.47	0.3	-0.18	0.27	0.03	15.15
0.34	-0.001	0.28	0.06	0.44	-0.11	0.36	0.08	13.62	0.38	-0.09	0.36	0.02	10.04	0.37	-0.12	0.33	0.04	11.14	0.39	-0.13	0.32	0.07	14.23
0.34	-0.01	0.28	0.06	0.45	-0.12	0.39	0.06	14.46	0.43	-0.11	0.36	0.07	12.95	0.49	-0.15	0.41	0.08	15.51	0.44	-0.12	0.36	0.08	16.04
0.34	-0.01	0.28	0.06	0.36	-0.09	0.35	0.01	11.76	0.4	-0.14	0.4	0.00	11.76	0.39	-0.15	0.37	0.02	12.4	0.51	-0.2	0.4	0.11	21.66
0.36	-0.03	0.3	0.06	0.34	-0.1	0.31	0.03	10.74	0.36	-0.12	0.36	0.00	10.54	0.39	-0.12	0.37	0.02	11.41	0.41	-0.14	0.38	0.03	13.34
0.33	0.00	0.27	0.06	0.37	-0.09	0.35	0.02	11.69	0.35	-0.12	0.33	0.02	10.89	0.37	-0.11	0.34	0.03	10.76	0.38	-0.15	0.32	0.06	15.52
0.34	0.00	0.28	0.06	0.29	-0.08	0.29	0.00	8.69	0.29	-0.12	0.31	-0.02	9.67	0.33	-0.14	0.32	0.01	10.71	0.32	-0.12	0.33	-0.01	9.85
0.34	0.00	0.28	0.06	0.36	-0.04	0.36	0.00	8.5	0.35	-0.08	0.37	-0.02	8.75	0.33	-0.07	0.34	-0.01	8.7	0.4	-0.1	0.35	0.05	12.62
0.34	0.00	0.28	0.06	0.29	-0.11	0.29	0.00	9.56	0.32	-0.13	0.33	-0.01	10.09	0.34	-0.11	0.33	0.01	10.14	0.32	-0.15	0.28	0.04	12.85
0.34	0.00	0.28	0.06	0.3	-0.08	0.031	0.269	10.04	0.4	-0.11	0.42	-0.02	9.14	0.32	-0.1	0.35	-0.03	9.03	0.4	-0.14	0.37	0.03	14.83
0.36	-0.03	0.3	0.06	0.24	-0.09	0.24	0.00	10.01	0.28	-0.11	0.28	0.00	10.37	0.27	-0.11	0.28	-0.01	10.66	0.34	-0.12	0.32	0.02	12.56
0.26	0.02	0.22	0.04	0.32	-0.09	0.34	-0.02	9.21	0.33	-0.08	0.35	-0.02	8.87	0.33	-0.08	0.35	-0.02	9.04	0.35	-0.08	0.36	-0.01	9.87
0.33	0.01	0.27	0.06	0.32	-0.09	0.33	-0.01	9.42	0.34	-0.11	0.34	0.00	10.55	0.35	-0.11	0.35	0.00	10.36	0.36	-0.14	0.32	0.04	13.55
0.33	0.00	0.27	0.06	0.33	-0.13	0.41	-0.08	14.06	0.43	-0.14	0.37	0.06	19.97	0.4	-0.15	0.32	0.08	18.76	0.47	-0.19	0.37	0.1	22.00
0.34	-0.01	0.28	0.06	0.25	-0.06	0.3	-0.05	7.56	0.29	-0.09	0.31	-0.02	9.13	0.29	-0.08	0.33	-0.04	8.61	0.31	-0.1	0.32	-0.01	10.6
0.35	-0.02	0.29	0.06	0.33	-0.1	0.33	0.00	10.39	0.37	-0.16	0.34	0.03	13.28	0.37	-0.14	0.33	0.04	11.2	0.41	-0.17	0.34	0.07	18.62
0.34	0.00	0.28	0.06	0.31	-0.13	0.31	0.00	11.05	0.32	-0.14	0.32	0.00	11.07	0.33	-0.14	0.33	0.00	10.45	0.35	-0.19	0.31	0.04	13.54
0.34	0.00	0.28	0.06	0.37	-0.1	0.4	-0.03	10.38	0.45	-0.17	0.36	0.09	16.03	0.39	-0.15	0.3	0.09	16.24	0.43	-0.18	0.4	0.03	20.44
0.34	-0.01	0.28	0.06	0.38	-0.1	0.38	0.00	9.97	0.53	-0.12	0.48	0.05	13.76	0.43	-0.12	0.43	0.00	9.63	0.43	-0.16	0.38	0.05	14.98
0.35	-0.02	0.29	0.06	0.33	-0.1	0.32	0.01	11.29	0.42	-0.15	0.4	0.02	11.67	0.39	-0.15	0.37	0.02	12.01	0.47	-0.2	0.42	0.05	18.09
0.36	-0.03	0.3	0.06	0.27	-0.08	0.3	-0.03	8.8	0.23	-0.08	0.27	-0.04	8.35	0.25	-0.1	0.28	-0.03	8.74	0.26	-0.12	0.26	0.00	11.16
0.34	-0.01	0.28	0.06	0.52	-0.16	0.39	0.13	24.7	0.51	-0.19	0.37	0.14	21.65	0.52	-0.2	0.36	0.16	19.59	0.51	-0.2	0.34	0.17	23.73
0.35	-0.02	0.29	0.06	0.33	-0.11	0.32	0.01	11.29	0.35	-0.15	0.36	-0.01	10.57	0.34	-0.15	0.34	0.00	10.84	0.33	-0.17	0.3	0.03	13.55
0.33	0.01	0.27	0.06	0.32	-0.12	0.3	0.02	10.64	0.32	-0.13	0.32	0.00	10.49	0.32	-0.13	0.32	0.00	10.32	0.34	-0.16	0.31	0.03	12.57

R5: total respiratory system resistance; **R20:** central airways resistance; **R5-R20**: peripheral airways resistance; **X5**: respiratory system reactance; **Fres:** resonant frequency.
